# Pan-Cancer Analysis on the Oncogenic Role of Programmed Cell Death 10

**DOI:** 10.1155/2022/1242658

**Published:** 2022-10-13

**Authors:** Ning Sun, Chenchen Li, Yue Teng, Yuxia Deng, Lin Shi

**Affiliations:** ^1^Department of Oncology, Jiangsu Cancer Hospital, Jiangsu Institute of Cancer Research, The Affiliated Cancer Hospital of Nanjing Medical University, Nanjing 210009, China; ^2^Department of Oncology, Zhongda Hospital Southeast University, Nanjing 210009, China

## Abstract

**Purpose:**

Programmed cell death factor 10 (PDCD10) is associated with intercellular junction, cytoskeleton organization, cell proliferation, apoptosis, exocytosis, and angiogenesis. However, the role of PDCD10 in human cancer is unclear. This study aims to explore the role of PDCD10 in various tumors and its possible mechanism through bioinformatics analysis.

**Methods:**

We verified the expression of the *PDCD10* gene based on data from the ONCOMINE, TIMER2.0, and TISDB databases. The correlation of PDCD10 with prognosis of patients with different tumors was analyzed using data from the GEPIA2 database. Proteins bound to PDCD10 were analyzed from the STRING database. PDCD10, PDCD10-binding proteins, and associated candidate genes were analyzed in DAVID for functional and pathway analyses. We also evaluated the immunological, clinical, and genetic aspects of distinct cancers by using TIMER2.0 and the connection between PDCD10 expression and tumor immune subtypes by using TISDB. Single-cell sequencing data from the CancerSEA database were used to characterize cancer cell functional states and generate heat maps.

**Results:**

PDCD10 overexpression is linked to certain molecular subtypes of human cancer. Low PDCD10 expression in patients with bladder urothelial carcinoma (BLCA), lung adenocarcinoma (LUAD), liver hepatocellular carcinoma (LIHC), adrenocortical carcinoma (ACC), head and neck squamous cell carcinoma (HNSC), kidney chromophobe carcinoma (KICH), brain lower grade glioma (LGG), pancreatic adenocarcinoma (PAAD), uterine corpus endometrial carcinoma (UCEC), oral squamous cell carcinoma (OSCC), and esophageal adenocarcinoma (ESAD) was correlated with favorable OS, whereas high PDCD10 expression in patients with LUSC, KIRC, READ, SKCM, and THYM was correlated with good prognosis. STRING network prediction results showed that 20 proteins, namely, paxillin (PXN), CCM2 scaffold protein (CCM2), TRAF3 interacting protein 3 (TRAF3IP3), FGFR1 oncogene partner 2 (FGFR1OP2), chromosome 4 open reading frame 19 (C4orf19), suppressor of IKBKE 1 (SIKE1), serine/threonine kinase 25 (STK25), striatin (STRN), protein phosphatase 2 catalytic subunit alpha (PPP2CA), mammalian sterile-20-like kinase 4 (MST4), MOB family member 4 (MOB4), protein phosphatase 2 scaffold subunit Abeta (PPP2R1B), sarcolemma-associated protein (SLMAP), serine/threonine kinase 24 (STK24), striatin 4 (STRN4), STRN3, protein phosphatase 2 scaffold subunit A alpha (PPP2R1A), striatin interacting protein 1 (STRIP1), CTTNBP2 N-terminal like (CTTNBP2NL), and cortactin binding protein 2 (CTTNBP2), can bind to PDCD10. Gene enrichment analysis suggested that PDCD10 is involved in the occurrence of different tumors through the Hippo signalling pathway, RNA transport, mRNA monitoring pathway, endocytosis, and T cell receptor signalling pathway. An inverse relationship was found between PDCD10 expression and cancer-associated fibroblasts in LUSC and TGCT, and PDCD10 expression was strongly connected with immunological subtypes, such as C1 (wound healing), C2 (interferon-gamma dominant), C3 (inflammation), C4 (lymphocyte depletion), C5 (immune silenced), and C6 (TGF-beta dominant). Finally, analysis of single-cell sequencing data revealed that PDCD10 expression is linked to epigenetic reprogramming, DNA repair, cell cycle progression, cell differentiation, inflammation, cell proliferation, cell differentiation, cell invasion, and angiogenesis.

**Conclusion:**

The results of our investigation demonstrate that PDCD10 has an oncogenic function in many cancer types. This study provides a reference for future research on antitumor therapeutic targets.

## 1. Introduction

The incidence of cancer is increasing worldwide, and the 2020 GLOBOCAN Cancer Statistics showed that the most commonly diagnosed cancers were lung cancer (22.1 million), breast cancer (2.26 million), and prostate cancer (14.1 million), and the most common causes of cancer-related death were lung cancer (1.79 million deaths), liver cancer (0.83 million deaths), and gastric cancer (769,000 deaths) [1]. Biomarkers are crucial to diagnose and predict the prognosis of cancer.

The programmed cell death factor 10 (PDCD10) gene is involved in programmed cell death. Physiological and pathological processes rely heavily on this intricate biological program. Members of the *PDCD* gene family show extensive genetic similarities and are broadly expressed. *PDCD* genes have been linked to cell death [2, 3], developmental problems, immunological illnesses, cancer, and other human diseases. PDCD10, which was first found in human premyeloid cells, is the gene activated in apoptotic cell death [4]. The N-terminal dimerization domain and the C-terminal focal adhesion targeting homology domain are the functional portions of the PDCD10 protein. The dimerization domain of PDCD10 has four helices, which are necessary for the protein to form homodimers [5, 6]. PDCD10 has also been linked to tumor formation and the initiation of other biological processes. Sun et al. [7] found that PDCD10 plays a role in the epithelial–mesenchymal transition (EMT) of HCC by directly binding to the protein phosphatase type 2A catalytic subunit (PP2Ac) and therefore enhancing PP2Ac enzymatic activity. Fu et al. [8] found that microRNA-103 suppresses tumor cell growth in prostate cancer by concentrating on PDCD10. PDCD10 has also been demonstrated to prevent apoptosis in malignant T cells while simultaneously promoting their proliferation [9]. In addition to its role in exocytosis and angiogenesis, PDCD10 has been involved in the formation of intercellular junctions and the structure of cytoskeleton. Nonetheless, the function of PDCD10 in malignancies remains unclear.

In the present study, we investigated PDCD10 expression and its predictive significance across various malignancies. By analyzing the functions of genes and protein-coding enzymes, we gained insights into the molecular processes by which PDCD10 and its binding proteins' function. The association between PDCD10 expression and immune infiltrating cells was then investigated. In addition, we used information from single-cell sequencing data to evaluate the state of cancer cells in relation to PDCD10.

## 2. Material and Methods

### 2.1. Pan-Cancer PDCD10 Gene Expression Analysis

Data were obtained from the ONCOMINE database (https://www.oncomine.org/resource/login.html) [10] and the TIMER2.0 website (http://timer.cistrome.org/) [11, 12]. The criteria for ONCOGENE expression are a *p* value <0.001 and a fold change of >2.0. The expression of PDCD10 was analyzed throughout cancer stages using the GEPIA2.0 database (http://gepia2.cancerpku.cn/#index) [13]. In addition, we analyzed the TISDB database (http://cis.hku.hk/TISIDB/index.php) [14] to compare PDCD10 expression in various molecular types of cancers.

### 2.2. Survival and Prognostic Analyses

From the GEPIA2 database, we extracted information on the overall survival (OS), PFS, and DFS of patients with various tumor types. Meanwhile, we classified PDCD10 expression levels into high and low groups. The log-rank test was then used to compare the survival rates of patients with various malignancies based on their PDCD10 expression.

### 2.3. Functional PDCD10 Enrichment

Using the STRING database (https://string-db.org/) [15], we investigated the proteins that interact with PDCD10. In addition, we selected the top 100 genes from the GEPIA2 database of genes with pan-cancer expression patterns comparable to PDCD10 as potential genes. PDCD10, PDCD10-binding proteins and 100 candidate genes were analyzed for GO enrichment and KEGG pathways in the DAVID database (https://david.ncifcrf.gov/) [16].

### 2.4. Immune Infiltration Analysis

To assess the immunological, clinical and genetic features of various tumors, TIMER2.0 offers a number of immune deconvolution techniques, including CIBERSORT, EPICa, and TIDE. Utilizing the TIMER2.0 database, we studied the connection between PDCD10 expression and immune cell infiltration in different malignancies and investigated the link between PDCD10 expression and cancer-associated fibroblasts (CAFs) in different cancers. Using the TISDB database, we also analyzed the correlation between PDCD10 expression and various immunological subtypes of tumors.

### 2.5. Analysis of Single-Cell Sequencing Data

We used CancerSEA (http://biocc.hrbmu.edu.cn/CancerSEA/home.jsp) [17], a single-cell sequencing database, to examine the various functional states of cancer cells. We grabbed the current state of CancerSEA's single-cell sequencing data and used it to generate a heat map illustrating the association between PDCD10 expression and several tumor functions. The CancerSEA database was used as the primary source for all single-cell t-SNE maps.

### 2.6. Statistical Analysis

The unpaired *t*-test was used to evaluate the statistical significance of the observed differences between the two groups, and the results were expressed as means ± standard deviations. The degree of relationship between the two groups was calculated using the Spearman correlation coefficient. Patients' survival rates were compared with their PDCD10 expression levels using the Kaplan–Meier technique. If the probability was less than 0.05, then the difference was considered significant.

## 3. Results

### 3.1. Expression of PDCD10 in Cancers

**Figure 1:**
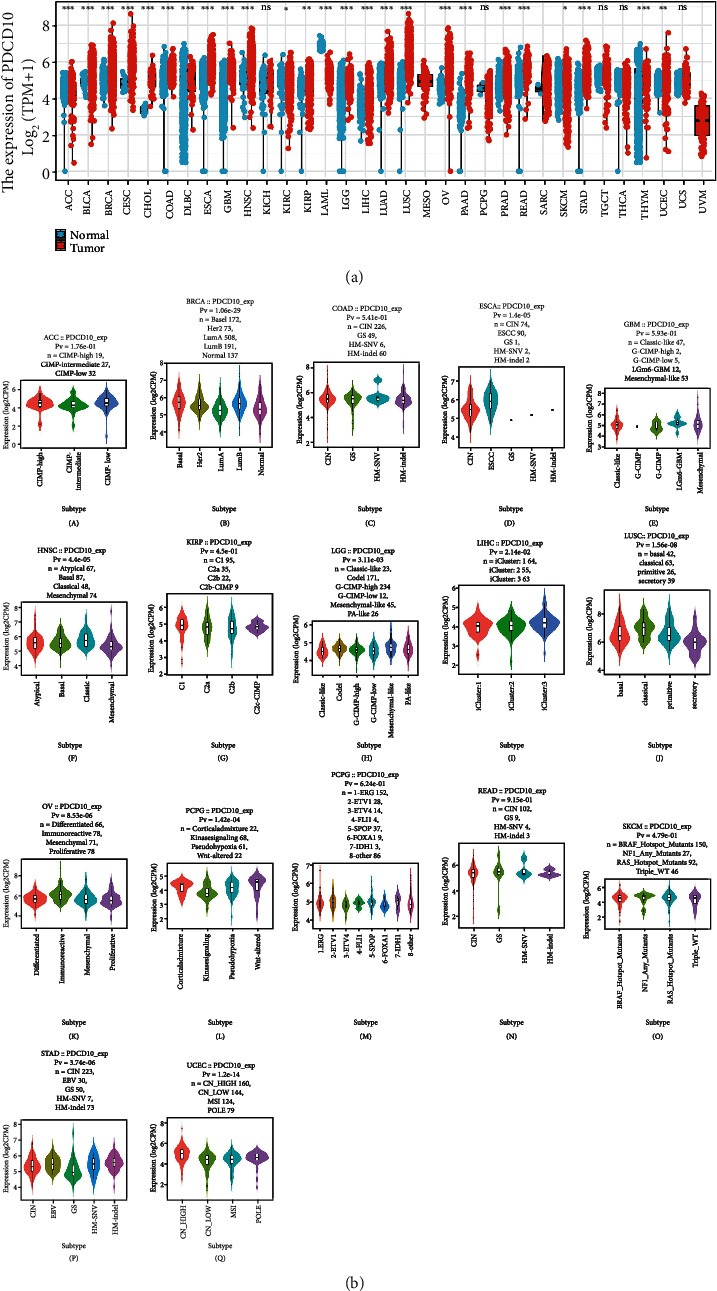
PDCD10 is overexpressed in some cancer types. PDCD10 expression in various cancers according to the TIMER2 database is shown in (a) (^∗∗^*p* < 0.01; ^∗∗∗^*p* < 0.001). (b) TISDB analysis of PDCD10 expression in ACC, BRCA, COAD, ESCA, GBM, and HNSC and in various molecular subtypes of KIRP, LUSC, LGG, LIHC, PCPG, OV, PRAD, READ, STAD, SKCM, and UCEC.

As a first step, we used the TIMER2.0 database to examine PDCD10 expression in cancers. PDCD10 was upregulated in acute myeloid leukemia (LAML) and thymoma (THYM) and downregulated in kidney chromophobe (KICH), pheochromocytoma and paraganglioma (PCPG), testicular germ cell tumors (TGCT), thyroid carcinoma (THCA), and uterine carcinosarcoma (UCS), and its expression was higher in cancer tissues than in neighboring tissues in most tumors (Figure 1(a)). We used the TISDB database to investigate the relationship of PDCD10 expression with a wide range of tumor molecular subtypes and discovered that it was positively correlated with esophageal carcinoma (ESCA), lung squamous cell carcinoma (LUSC), and stomach adenocarcinoma (STAD) (*p* < 0.05; Figure 1(b)). PDCD10 expression was higher in several tumor tissues than in normal tissues, indicating that PDCD10 may act as an oncogene in these tumors, which is consistent with previous findings [18, 19].

### 3.2. PDCD10 in the Prognosis of Various Cancers

**Figure 2:**
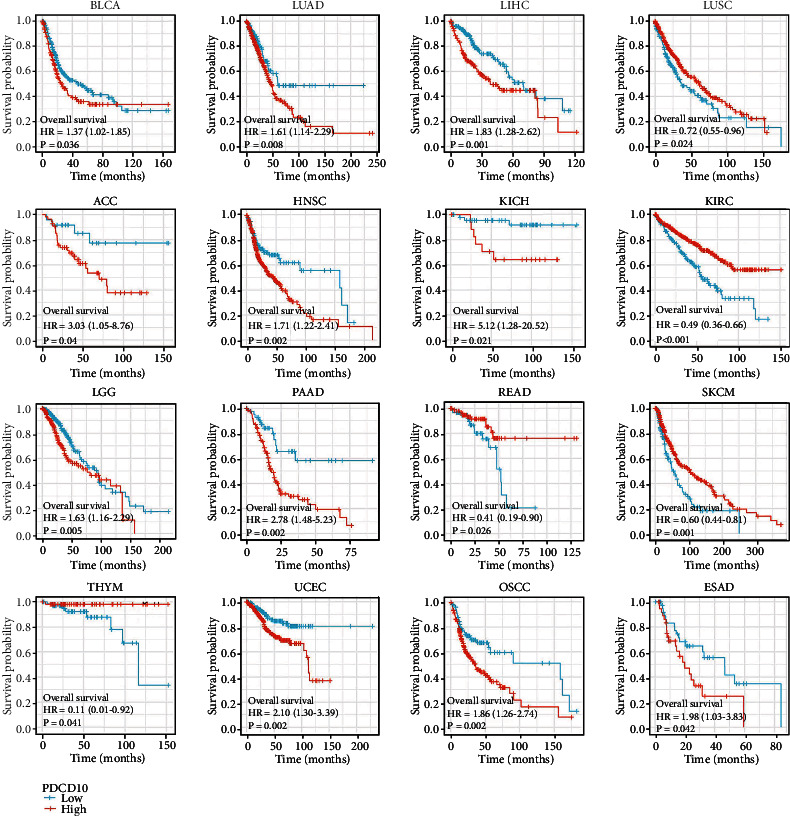
Prognostic value of PDCD10 expression in different cancer types. The relationship between PDCD10 expression and OS in different tumors from the TCGA database was studied using the GEPIA2 database (only the positive data of Kaplan–Meier curves are shown).

**Figure 3:**
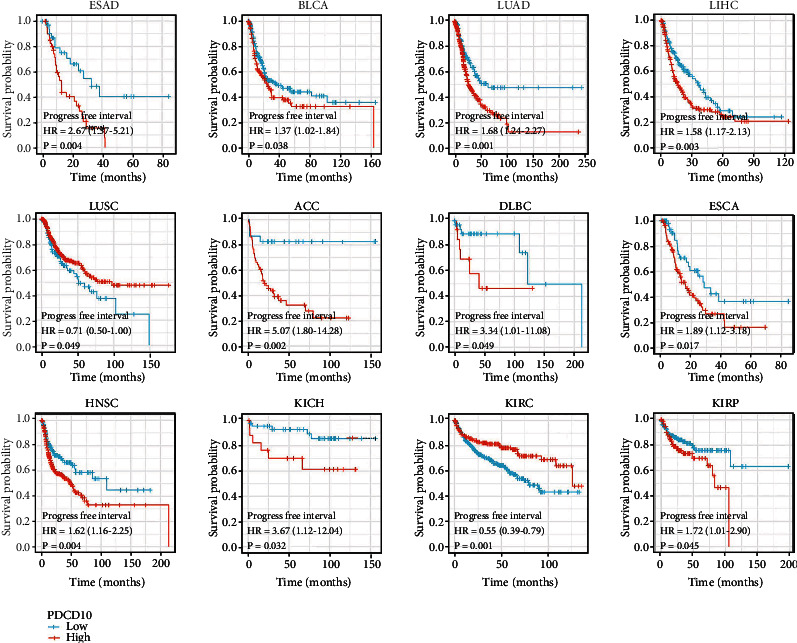
Prognostic value of PDCD10 expression in different cancer types. The relationship between PDCD10 expression and PFI in different tumors from the TCGA database was studied using the GEPIA2 database (only the positive data of Kaplan–Meier curves are shown).

**Figure 4:**
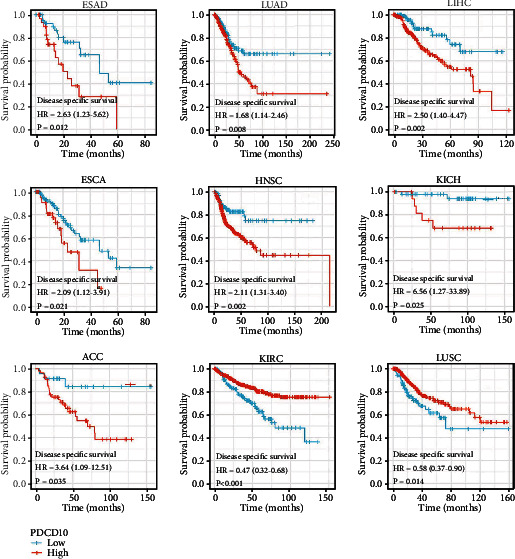
Prognostic value of PDCD10 expression in different cancer types. The relationship between PDCD10 expression and DSS in different tumors from the TCGA database was studied using the GEPIA2 database (only the positive data of Kaplan–Meier curves are shown).

We conducted a survival analysis using the GEPIA2 database to investigate whether or not PDCD10 expression is associated with a better or worse outcome for patients with various tumor types. Results showed that low PDCD10 expression in patients with BLCA, LUAD, LIHC, ACC, HNSC, KICH, LGG, PAAD, UCEC, OSCC, and ESAD correlated with high OS rates, whereas high PDCD10 expression in patients with LUSC, KIRC, READ, SKCM, and THYM correlated with good prognosis (Figure 2). In addition, low PDCD10 expression in patients with ESAD, BLCA, LUAD, LIHC, ACC, DLBC, ESCA, HNSC, KICH, KIRP, LGG, PAAD, UCEC, OSCC, and PDCD10 correlated with high progression-free interval (PFI) (Figure 3). In addition, low PDCD10 expression in patients with LUSC, KIRC, READ, SKCM, and THYM was linked to low disease-specific survival (DSS) rates (Figure 4).

### 3.3. Co-Expression Network and Pathway Analysis of PDCD10

**Figure 5:**
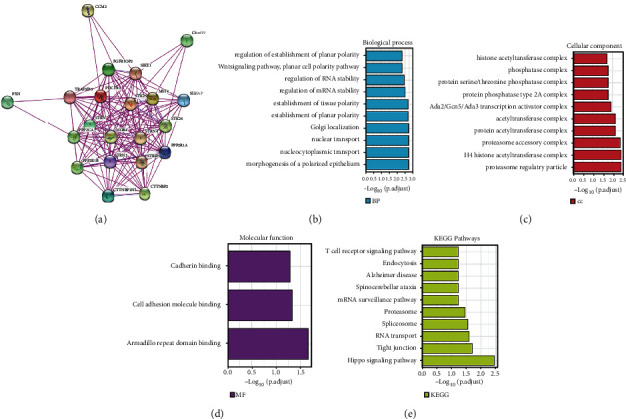
Enrichment pathways and co-expression network of PDCD10. (a) Biological process, (b) cellular component, (c) molecular function, (d) PDCD10-binding protein recognized by STRING database, and (e) KEGG pathway based on PDCD10-binding proteins and associated genes.

Based on the findings, we hypothesized that PDCD10 is an oncogene in many cancer types and thus may be employed as a prognostic marker. However, the precise molecular mechanism through which PDCD10 contributes to cancer remains unknown. PDCD10 binding proteins were predicted using the STRING network, and then, its co-expression network and enrichment pathways in many cancer types were investigated. Results showed that 20 proteins, namely, paxillin (PXN), CCM2 scaffold protein (CCM2), TRAF3 interacting protein 3 (TRAF3IP3), FGFR1 oncogene partner 2 (FGFR1OP2), chromosome 4 open reading frame 19 (C4orf19), suppressor of IKBKE 1 (SIKE1), serine/threonine kinase 25 (STK25), striatin (STRN), protein phosphatase 2 catalytic subunit alpha (PPP2CA), mammalian sterile-20-like kinase 4 (MST4), MOB family member 4 (MOB4), protein phosphatase 2 scaffold subunit Abeta (PPP2R1B), sarcolemma-associated protein (SLMAP), serine/threonine kinase 24 (STK24), striatin 4 (STRN4), STRN3, protein phosphatase 2 scaffold subunit A alpha (PPP2R1A), striatin interacting protein 1 (STRIP1), CTTNBP2 N-terminal like (CTTNBP2NL), and cortactin binding protein 2 (CTTNBP2), can bind to PDCD10 (Figure 5(a)). From GEPIA2, we compiled a list of the top 100 genes most strongly associated with PDCD10. Then, we analyzed the genes in each collection using GO and KEGG enrichment analyses. Enrichment indicated regulation of these genes with mRNA stability, planar cell polarity pathway, Wnt signalling pathway, Golgi localization, nucleocytoplasmic trafficking, nuclear trafficking, Ada2/Gcn5/Ada3 transcriptional activator complex, H4 histone acetyltransferase complex, type 2A protein phosphatase complex, protein serine/threonine phosphatase complex, phosphatase complex, cell adhesion molecule binding, and cadherin binding associated (Figures 5(b)–5(d)). In addition, Hippo signalling, RNA transport, mRNA surveillance pathway, endocytosis, and the signalling of T cell receptors were involved in the tumorigenic effects of PDCD10 (Figure 5(e)).

### 3.4. PDCD10 Expression Was Negatively Correlated with CAF

**Figure 6:**
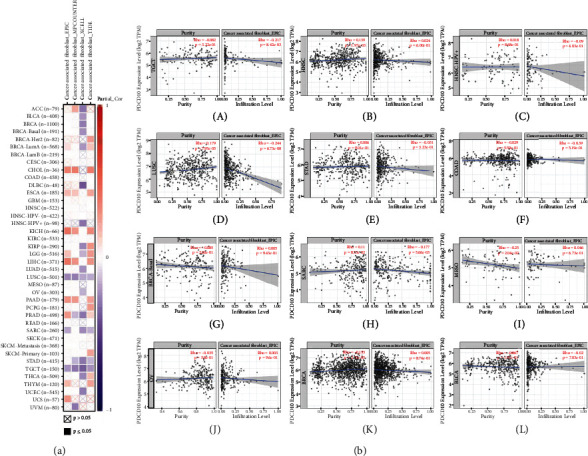
(a) and (b) show the link between PDCD10 expression and tumor-associated fibroblasts based on the TIMER2 database.

**Figure 7:**
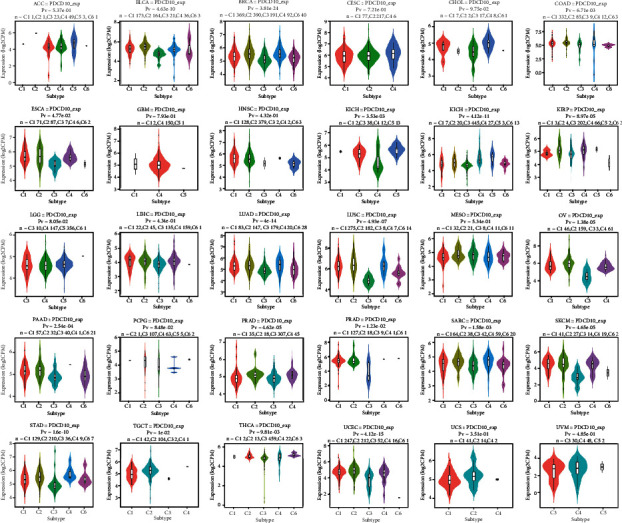
PDCD10 expression levels in various cancer immune subtypes based on the TISDB database.

The link between tumor cells and their surrounding environment is provided by the tumor microenvironment (TME). The TME has four primary parts: immune system, blood vessels, extracellular matrix, and stroma. Many types of immune cells, including T cells and B cells, comprise the immune system, and colony-forming units and mesenchymal stem cells comprise the stromal component [20, 21]. The centrality of CAFs in cancer development and immune response has been highlighted by recent studies. For example, Wanandi et al. [22] found that CAF secretome induces EMT in HT-29 colorectal cancer cells via hepatocyte growth factor signalling. Similarly, Zarin et al. [23] reported that CAF and CAF-derived exosomes are crucial to the development and spread of malignancies in the digestive system. In addition, fibroblasts play a role in cancer. microRNA-148b-3p, when expressed from exosomes and downregulated, increases PTEN expression and decreases Wnt/*β*-catenin pathway activity, which contribute to the chemotherapy resistance of bladder cancer cells [24]. Using the TIMER 2.0 database, we compared the expression of PDCD10 with those of other TME components in many cancer types to understand the function of PDCD10 in the TME. The data of EPIC, MCPCOUNTER, XCELL, and TIDE showed that PDCD10 had an inverse relationship with CAF in LUSC and TGCT (Figure 6(a)) and that CAFs in SARC, MESO, OV, BRCA, and BLCA were all positively linked with one another (Figure 6(b)). We investigated CAF expression markers in various cancer types to elucidate the mechanisms underlying the link between PDCD10 expression and CAF. The expression of PDCD10 was strongly correlated with immunological subtypes, such as C1–C6 (Figure 7).

### 3.5. Single-Cell PDCD10 Expression and Cancer Functional State

**Figure 8:**
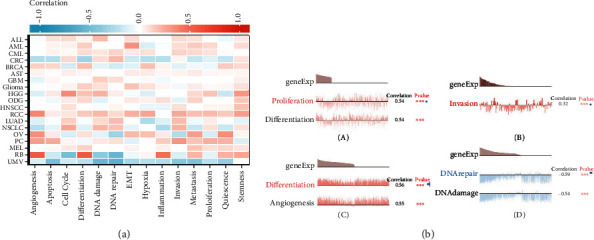
Expression patterns of PDCD10 in single cells and how they relate to the functional condition of the tumor. Based on the CancerSEA database, (a) a heat map illustrating the relationship between PDCD10 expression and functional status of various malignancies (^∗^*p* < 0.05). (b) Correlation between four substantially distinct functional states as determined by the CancerSEA database and PDCD10 expression (^∗^*p* < 0.05).

**Figure 9:**
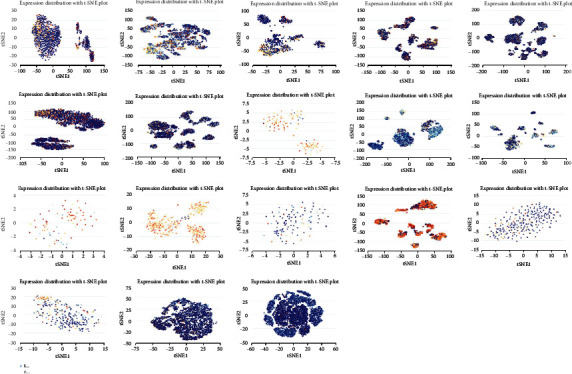
T-SNE plot demonstrating the expression of PDCD10 in single cells from samples of AML, CML, GBM, glioma, AST, HGG, ODG, LUAD, NSCLC, MEL, RCC, BRCA, PC, HNCC, OV, CRC, RB, and UM.

Single-cell transcriptome sequencing is an important method for studying different types of cancer, immune, endothelial, and stromal cells [25, 26]. PDCD10 expression in AML was strongly positively connected with EMT, which agree with previous studies that have linked PDCD10 expression to tumor functional state. PDCD10 showed a strong positive association with DNA repair in CRC, cell cycle in HGG, and differentiation and inflammation in RB; meanwhile, it showed a significant negative correlation with DNA repair in RB and UM and DNA damage repair (Figure 8(a)). As shown in Figure 8(b), PDCD10 expression is associated with cell proliferation and differentiation in AML, invasion in PC, differentiation and angiogenesis in RB, and PDCD10 expression in UM and DNA damage repair.  Figure 9 illustrates the T-SNE plots of PDCD10 expression patterns in AML, CML, GBM, glioma, AST, HGG, ODG, LUAD, NSCLC, MEL, RCC, BRCA, PC, HNCC, OV, CRC, RB, and UM single cells (Figure 9). The foregoing data all imply that PDCD10 plays a major role in the biological processes of tumor incidence and development.

## 4. Discussion

A pan-cancer analysis is useful for discovering biomarkers for early cancer diagnosis and targeted treatment because it provides comprehensive information on molecular abnormalities in different tumors. Bermez-Guzmán et al. [27] conducted a pan-cancer analysis and provided evidence for the presence and significance of a nononcogenic addiction to DNA repair in cancer, which assisted in the identification of prognostic biomarkers and treatment options. Secreted frizzled-related proteins (SFRPs) consisting of five family members (SFRPs1–5) were postulated to be extracellular Wnt inhibitors by Vincent and Postovit [28]. Some studies have also found that the methylation signature of cancer immunotherapy response can be predicted by pan-cancer analysis [29]. Park et al. [30] proposed that pan-cancer methylation analysis could reveal a negative correlation between tumor immunogenicity and methylation abnormalities, highlighting the significance of methylation abnormalities for tumors to evade immune surveillance and aiding in the development of methylation biomarkers. A pan-cancer investigation of solid tumor genomes found no discernible variations between metastatic and original tumor genomes in terms of mutational patterns or driver genes [31]. Prostate cancer growth is aided by CircSMARCA5 through the miR-432–PDCD10 axis [32]. PDCD10 promotes cell proliferation and transformation by regulating the extracellular signal-regulated kinase (ERK) pathway [33]. PDCD10 interacts with the Ste20-related kinase MST4. Due to PDCD10 deficiency, glioblastoma cells become activated and facilitate tumor development [34]. We carefully examined the expression and prognostic significance of PDCD10 in various cancer types to understand its function in cancer development. From these data, we demonstrate that PDCD10 is highly expressed in tumors than in the comparable paraneoplastic tissues. These findings suggest that PDCD10 is an oncogene in these malignancies.

Previous pan-cancer analyses have uncovered the significance of aberrantly expressed genes in the onset and/or progression of colorectal cancer; for instance, ARID1A alterations impair mismatch repair pathways and increase the number of tumor-infiltrating lymphocytes and PD-L1 expression in gastric cancer. Therefore, ARID1A might interact with ICIs in the treatment of stomach cancer [35]. A pan-cancer investigation revealed that the expression of NOS3 correlates with the response of STAD to QS-11 and brivinib [36], indicating the importance of the protein in the treatment of gastric cancer.

At present, researchers are increasingly focusing on the TME [37]. Given its central role in the TME, CAF plays a number of tumor-promoting roles throughout carcinogenesis and development. For example, TGF- controls the invasion of ovarian cancer by increasing CAF-derived versican [38]. Hepatocellular carcinoma has a dismal prognosis due to the osteopontin pathway, which mediates communication between cancer-associated fibroblasts and tumor-associated macrophages in TME [39]. Our results suggest that PDCD10 expression is inversely associated with CAF in various malignancies, but further research is needed to determine the precise mechanism by which this correlation is mediated. Recent immunogenomic research of 33 distinct cancer types [40] revealed six immunological subtypes (C1, C2, C3, C4, C5, and C6) for the first time. Many heterogeneous cancers can be differentiated easily by the discovery of novel immune subtypes, which may help with the tailored immunotherapy of patients with cancer. Our data showed that PDCD10 expression was highly linked with the immunological subtypes of many cancer types, including PC, OV, CRC, and STAD, suggesting that this protein plays a crucial role in cancer immunotherapy.

Single-cell transcriptome sequencing analysis revealed a strong positive correlation between PDCD10 in AML and EMT. CRC PDCD10 showed a strong positive association with DNA repair. A substantial positive correlation was also found between PDCD10 and cell cycle in HGG. In RB, PDCD10 exhibited strong positive correlations with differentiation and inflammation and strong negative correlations with DNA repair. DNA damage repair was strongly adversely linked with PDCD10 in UM.

## 5. Conclusion

We analyzed PDCD10 expression in various tumor types and discovered that it is upregulated in malignancies and inversely connected with CAF. We revealed the potential role of PDCD10 as a prognostic indicator for patients with different tumor types and its potential role in affecting tumor immunotherapy efficacy by affecting TME. This study could serve as a reference for the development of PDCD10 into a therapeutic target in cancers in the future.

## Data Availability

All experimental data used to support the findings of this study are available from the corresponding author upon request.
